# Pancreatic Ductal Adenocarcinoma Cells Regulate NLRP3 Activation to Generate a Tolerogenic Microenvironment

**DOI:** 10.1158/2767-9764.CRC-23-0065

**Published:** 2023-09-20

**Authors:** Jesus Amo-Aparicio, Adrian Dominguez, Shaikh M. Atif, Alberto Dinarello, Tania Azam, Kibrom M. Alula, Miles Piper, Christopher H. Lieu, Robert W. Lentz, Alexis D. Leal, Stacey M. Bagby, Wells A. Messersmith, Sana D. Karam, Charles A. Dinarello, Todd M. Pitts, Carlo Marchetti

**Affiliations:** 1 Department of Medicine, University of Colorado Anschutz Medical Campus, Aurora, Colorado.; 2 Department of Medicine, Division of Medical Oncology, University of Colorado Anschutz Medical Campus, Aurora, Colorado.; 3 Department of Radiation Oncology, University of Colorado Denver-Anschutz Medical Campus, Aurora, Colorado.

## Abstract

Defining feature of pancreatic ductal adenocarcinoma (PDAC) that participates in the high mortality rate and drug resistance is the immune-tolerant microenvironment which enables tumors to progress unabated by adaptive immunity. In this study, we report that PDAC cells release CSF-1 to induce nucleotide-binding domain, leucine-rich containing family, pyrin domain-containing-3 (NLRP3) activation in myeloid cells. Increased NLRP3 expression was found in the pancreas of patients with PDAC when compared with normal pancreas which correlated with the formation of the NLRP3 inflammasome. Using human primary cells and an orthotopic PDAC mouse model, we show that NLRP3 activation is responsible for the maturation and release of the inflammatory cytokine IL1β which selectively drives Th2-type inflammation via COX2/PGE2 induction. As a result of this inflammation, primary tumors were characterized by reduced cytotoxic CD8^+^ T-cell activation and increased tumor expansion. Genetic deletion and pharmacologic inhibition of NLRP3 enabled the development of Th1 immunity, increased intratumoral levels of IL2, CD8^+^ T cell–mediated tumor suppression, and ultimately limited tumor growth. In addition, we observed that NLRP3 inhibition in combination with gemcitabine significantly increased the efficacy of the chemotherapy. In conclusion, this study provides a mechanism by which tumor-mediated NLRP3 activation exploits a distinct adaptive immunity response that facilitates tumor escape and progression. Considering the ability to block NLRP3 activity with safe and small orally active molecules, this protein represents a new promising target to improve the limited therapeutic options in PDAC.

**Significant:**

This study provides novel molecular insights on how PDAC cells exploit NLRP3 activation to suppress CD8 T-cell activation. From a translational perspective, we demonstrate that the combination of gemcitabine with the orally active NLRP3 inhibitor OLT1177 increases the efficacy of monotherapy.

## Introduction

Pancreatic ductal adenocarcinoma (PDAC) constitutes 90% of all pancreatic cancers and it is the third leading cause of cancer deaths in the United States and seventh worldwide (1, 2). Lack of effective screening methods, nonspecific symptoms and limited treatment options are major limitations in the management of this disease that contribute to the extremely severe prognosis in patients with pancreatic cancer (the 5-year survival reached 11% for the first time in 2022). Considering the very modest impact of the standard of care on the overall survival of patients with advance disease, novel treatment options represent a medical urgency for these patients.

Inflammation has been demonstrated to influence PDAC growth, response to therapy, and metastasis with several inflammatory cytokines found increased in patients with PDAC (3–5). Of these, the inflammatory cytokine IL1β has been shown to be upregulated in human PDAC tissue, to promote desmoplasia, immune suppression, and to correlate to patient's response to chemotherapy (6–8). IL1β is initially synthesized as a biologically inactive pro-form which requires processing and releasing (9). Maturation of IL1β is mostly regulated by conserved cytosolic pattern recognition receptors of the nucleotide-binding and oligomerization domain NOD-like receptor (NLR) family (10). Once activated, NLRs form cytosolic macromolecular complexes called “inflammasomes” which are responsible for the conversion of biologically inactive IL1β and IL18 precursors into their active forms via caspase-1 cleavage (10). In the context of the pancreas, NLRP3, one NLRs member, has shown to mediate inflammation in acute pancreatitis, a recognized high-risk factor for the development of PDAC (11). Furthermore, NLRP3 signaling has shown to participate in tumor progression in a mouse model of PDAC and to correlate with reduced survival in patients with pancreatic adenocarcinoma (12, 13). Albeit these observations suggest a key role for NLRP3 in PDAC, the mechanism of its activation and biological function in PDAC remain unclear. In this study, we report a novel PDAC-mediated inflammatory network that mechanistically links CSF-1 production by PDAC cells to NLRP3 activation in the tumor microenvironment (TME). Herein, we also show that NLRP3 activation promotes Th2 polarization, leading to immune suppression and tumor progression. From a translational perspective, we report that pharmacologic inhibition of NLRP3 with the human-safe and orally active NLRP3 inhibitor OLT1177 (INN dapansutrile) increases the efficacy of gemcitabine, a chemotherapeutic agent used for the treatment of advanced PDAC. In conclusion, these studies provide evidence that PDAC cells exploit NLRP3 activation to generate a tolerogenic TME and its inhibition could represent a valid option to increase the efficacy of standard of care.

## Materials and Methods

### Cell Culture

MiaPaca-2, Panc-1, and AsPC-1 cell lines were maintained in DMEM supplemented with 10% FBS, 1% nonessential amino acids (Gibco), and 1% penicillin-streptomycin. Cells were seeded at 3,000 cells per well in a 96-well plate in 200 µL. Supernatant and lysates were collected at 24, 48, and 72 hours after seeding. For conditioned medium (CM), cells were seeded at 150,000 cells per well in a 12-well plate. After 72 hours, supernatants were collected and centrifuged for 4 minutes at 244 × *g*. THP-1 and THP-1 *NLRP3*^−/−^ cells were seeded at 100,000 cells per well in a 96-well plate in 200 µL and stimulated with CM for 72 hours.

Peripheral blood mononuclear cells (PBMC) were obtained from the venous blood of healthy donors. Cells were isolated using centrifugation in a Histopaque-1077 (Sigma-Aldrich) gradient. Collected cells were counted using a hemocytometer (Heska), then centrifuged for 4 minutes at 244 × *g* and resuspended in RPMI1640 supplemented with 10% FBS and 1% penicillin-streptomycin. 100,000 cells per well were seeded in a 96-well plate in 200 µL and stimulated with CM for 72 hours.

#### Public Database and Gene Expression Analysis

Normalized gene expression data from The Cancer Genome Atlas (TCGA) project were downloaded from the gene expression profiling interactive analysis.

#### *In Vivo* Model

Animal protocols were approved by the University of Colorado Animal Care and Use Committee. Eight weeks old C57BL/6 and *Nlrp3*^−/−^ male mice were purchased from Jackson Laboratories. Eight weeks old NCG male mice were purchased from Charles Rivers. For orthotopic pancreatic tumor challenge, mice received an intrapancreatic injection of FC1242 tumor cells derived from KPC mice backcrossed with C57BL/6 background (14). Cells were kindly provided by Dr. S.D. Karam through an agreement with Dr. Tuveson, who generated them. FC1242 cells were plated for 2 days in RPMI1640 (Corning) with 10% (v/v) FBS (Corning) and 1% penicillin-streptavidin (Corning). Cells were collected and suspended in 1:1 solution with RPMI1640 medium containing 1% penicillin-streptavidin and Matrigel (Corning). A total of 1 × 10^5^ cells in 20 µL were injected into the body of the pancreas via laparotomy. Mice were sacrificed at 14 or 21 days after the surgery, and tumor dimensions and weight were recorded. Tumor volumes were calculated as described previously (15). For survival, 2 × 10^5^ cells were injected following the same protocol. Natural death and humane endpoints were applied to collect datapoints following the guidelines from the University of Colorado (Aurora, CO).

#### Reagents and Treatments

For *in vitro* experiments, CM was used at 50% dilution to stimulate THP-1 and PBMCs for 72 hours. Dapansutrile (lab code: OLT1177) was provided by Olatec Therapeutics LLC and was prepared fresh every time and used at 10 µmol/L. MCC950 (Tocris) was used at the same concentration. Human α-CSF1R (CD115, BioXcell) was used at 10 and 1 µg/mL. AZD7507 (MedChemExpress) was used at 1 µmol/L. Reagents were added to THP-1 or PBMCs culture 1 hour before CM. For blockade of CSF-1 and IL34 in the CM, neutralizing antibodies against human α-CSF-1 and α-IL34 (R&D Systems) were used at 10 µg/mL and incubated overnight at 4°C. Matching IgG was used as control. For *in vivo* experiments, OLT1177 was supplied *ad libitum* using OLT1177-fortified food pellets (7.5 g/kg) for the entire duration of the experiment starting after the surgery. Control mice received a matching standard diet. Mice were monitored during the study for any changes in physiologic parameters, including irregular weight gain or loss as well as changes in body temperature. Mouse α-CSF1R (BioXcell) or a matching IgG were administrated by intraperitoneal injection at 75 mg/kg every 3 days until the end of the experiment starting on day 1 after surgery as described previously (16). Gemcitabine (Hospira) was administrated intraperitoneally at 50 mg/kg every 3 days starting 10 days after surgery as described previously (17).

### Organoid Viability

PDAC biopsy samples were digested with TrypLE solution to achieve a single-cell suspension. Cells are diluted at 1.3 × 10^5^ cells/mL in basement membrane extract (BME, Trevigen) and plated into 96-well plates (15 µL/well). Organoid domes were maintained in Human Pancreatic Stem Cell medium which contains 50% WRN CM (Wnt-3a, R-Spondin, and Noggin), B27supplement, NAC, Nicotinamide, 50 ng/mL human EGF, 500 nmol/L A83-01 (ALK inhibitor), 10 nmol/L human (Leu15)-Gastrin I, 100 ng/mL human FGF10, and 100 mg/mL puromycin, and 10 µmol/L Y27632. Following 7 days, OLT1177 (1 and 10 µmol/L) and gemcitabine (100 nmol/L; Hospira) were added to the mature organoids. Organoid viability was assayed after 72 hours from the drug treatments using CellTiter Glo three-dimensional growth assay (Promega) according to the manufacturer's instructions. Results were normalized to the vehicle (PBS).

#### Flow Cytometry

Tumors were isolated and digested using 1% Liberase DL and 1% of Liberase TL (Sigma-Aldrich) at 37**°**C for 45 minutes. Enzymatic disaggregation was blocked by adding PBS with 1%w/v of BSA (Sigma-Aldrich). Tumors were then homogenized using a 70 µm cell strainer. Single-cell suspensions were stained using: CD3-PE, CD4-BV785, CD8e-APC/Cy7, CD45-BV510, CD44-FITC, PD1-PERCP, CD62L-BV650, CD11b-BV785, Ki67-APC. Antibodies were purchased from BioLegend and used following manufacturer recommendations. Cells were analyzed using the BD LSRFortessa flow cytometer (BD Biosciences). Gating and quantification were performed using FlowJo software.

#### Protein Analysis

Tumor samples were homogenized in RIPA buffer (Thermo Fisher Scientific) supplemented with protease inhibitor (Thermo Fisher Scientific) using a TissueRuptor (Qiagen). Protein concentration was determined using a bicinchoninic acid Protein Assay Kit (Thermo Fisher Scientific) according to the manufacturer's instructions. Samples were diluted to 2 mg/mL.

Levels of mouse COX-2 and PGE2 from tumors were measured by ELISA kits (R&D Systems) according to the manufacturer's directions. Results were measured in a microplate reader (Bio-Tek). All cytokine levels for *in vitro* and *in vivo* experiments were measured by specific DuoSet ELISAs according to the manufacturer's instructions (R&D Systems).

#### PyK2 Assay

THP-1 cells were stimulated with PDAC-conditioned media in the presence and absence of α-CSF-1 and α-IL34 antibodies as described previously. Phosphorylation of PyK2 was measured in the cell lysate using Proteome Profiler (R&D Systems, Inc.) according to the manufacturer's protocol.

#### Western Blotting

Cell lysates were resolved on a Mini-PROTEAN TGX 4–20% gel (Bio-Rad) and transferred to a 0.1 µmol/L nitrocellulose membrane (GE Healthcare). Membranes were blocked in 5% blocking buffer (Bio-Rad) in PBS-T 0.5% for 1 hour at room temperature. Samples were incubated with primary antibody for NLRP3 (1:1,000, AdipoGen). Peroxidase-conjugated secondary antibodies (Jackson ImmunoResearch) and chemiluminescence were used to detect the protein concentration. Conjugated antibody against β-actin (1:1,000, Santa Cruz Biotechnology) was used to normalize protein concentrations.

#### RNA Extraction and Gene Measurement

Tumor samples were homogenized in TRIzol reagent (Thermo Fisher Scientific) using a TissueRuptor (Qiagen). RNA was extracted using chloroform and isopropanol. RNA was reversely transcribed using a High-Capacity cDNA Reverse Transcription kit (Applied Biosystems). qPCR was performed using a PowerUp SYBR Green Master Mix (Applied Biosystems) in a QuantStudio 3 Real-Time PCR System (Applied Biosystems). Primers for mouse *Il2*f5′-GCGGCATGTTCTGGATTTGACTC-3′, *Il2*r5′-CCACCACAGTTGCTGACTCATC-3′, *Il4*f5′-CATGGGAAAACTCCATGCTT-3′, *Il4r*5′-TGGACTCATTCATGGTGCAG-3′, *Il10f*5′-CGGGAAGACAATAACTGCACCC-3′, *Il10r*5′-CGGTTAGCAGTATGTTGTCCAGC-3′, *Ifng*f5′-CAGCAACAGCAAGGCGAAAAAGG-3′, *Ifng*r5′-TTTCCGCTTCCTGAGGCTGGAT-3′, *Prf1*f5′-TCTTGGTGGGACTTCAGCTT-3′, *Pfr1*r5′-TGCTTGCATTCTGACCGAGT-3′ were used. *Gapdh* was used as a housekeeping gene with the primers *Gapdh*f 5′-TTCAACAGCAACTCCCACTCTTCCA-3′ and *Gapdh*r5′-ACCCTGTTGCTGTAGCCGTATTCA-3′. Expression levels of target mRNAs were normalized to the relative ratio of expression of the *Gapdh* gene and standard-diet condition following the ΔΔCT method.

#### Histology and Immunofluorescence

Mouse tumor samples were collected and kept in 4% paraformaldehyde (Sigma-Aldrich) in PBS. After dehydration, samples were embedded in paraffin molds for sectioning. Sections (5 µm) were obtained and transferred to glass microscope slides. Slides were deparaffinized with xylene and ethanol and permeabilized with 0.1% Tween in TBS solution. After antigen retrieval with citrate buffer, slides were blocked with 10% donkey serum and incubated with primary antibodies against NLRP3 (rat, R&D Systems) and ASC (goat, Abcam) at 1:100 dilution overnight at 4°C. Alexa flour 647 anti-rat and Alexa fluor 555 anti-goat at 1:100 (Invitrogen) were used as secondary antibodies for 1 hour at room temperature. Finally, slides were coverslipped in Mowiol mounting media with DAPI. Images were taken using Olympus FV1000 laser scanning confocal/CARS microscope. Colocalization was performed using NIH ImageJ software. For human biopsy samples, same protocol was used except for ASC (rabbit, Adipogen) and Alexa Flour 488 antibodies (Invitrogen). For IHC analysis, primary tumor sections were stained using anti-IL1β (p17; Cell Signaling Technology). Processed IL1β (p17) immunoreactivity was calculated by measuring the integrated density by NIH ImageJ software.

### ASC Specks Quantification

ASC immunofluorescence staining was analyzed using the Fiji (ImageJ) software. Briefly, after splitting the RGB channels, a threshold was set for specks channel to remove the background. The same threshold was used for all the pictures analyzed. Specks were analyzed using the “Analyze particles” command. Similar strategy was used to count the nuclei in the DAPI channel. The resulting specks count was divided by the total nuclei count of each respective picture to obtain an estimate of the number of specks per cell. At least five pictures per sample were analyzed to quantify the ASC specks.

### Statistical Analysis

All the analyses were conducted by GraphPad Prism 10 software. Statistical significance of differences was evaluated with a two-tailed Student *t* test. For data containing three or more groups, one-way ANOVA with Dunnett or Turkey *post hoc* correction was used. For CD62L/CD44 populations, two-way ANOVA with Bonferroni *post hoc* correction was used. Wilcoxon matched-pairs signed rank test was used to measure cytokine increase from freshly isolated PBMCs after CM stimulation. Kaplan–Meier test was used for survival. All quantitative data are presented as the means ± SEM. A *P* value of less than 0.05 was considered significant.

### Data Availability Statement

The data generated in this study are available upon request from the corresponding author.

## Results

### NLRP3 Activation is Increased in PDAC Samples

**FIGURE 1:**
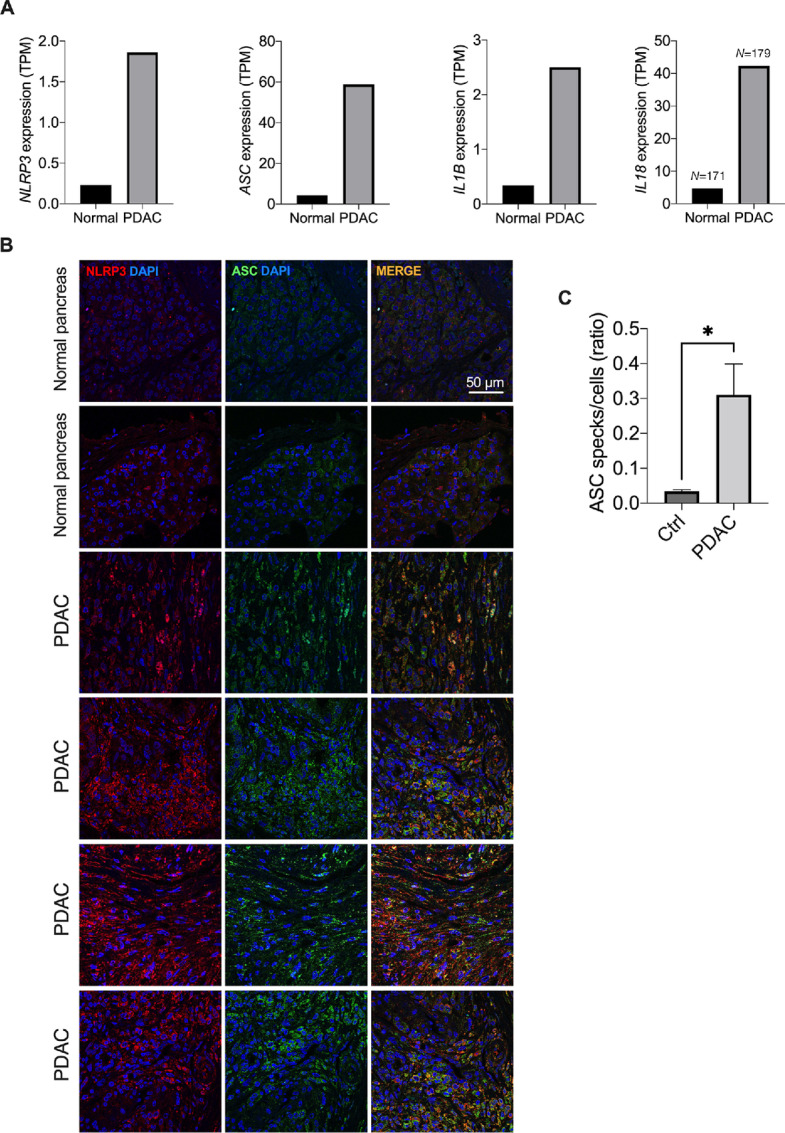
NLRP3 expression and activation are increased in PDAC biopsy. **A,** NLRP3, ASC, IL1β, and IL18 median expression levels in human PDAC biopsy and normal pancreas samples (TCGA dataset). **B,** Immunofluorescence staining from NLRP3 (red) and ASC (green) in biopsy of normal pancreas (*n* = 2) and patients with PDAC (*n* = 4). Each row represents a single patient. **C,** Quantification of ASC specks–like structure samples in B. *, *P* < 0.05.

NLRP3 gene expression was found elevated in the pancreas of patients with pancreatic adenocarcinoma (*n* = 179) when compared with normal tissue (*n* = 171; Fig. 1A). Similar increase was found in genes associated with NLRP3 activation such as apoptosis-associated speck-like protein containing a CARD (ASC), IL1β, and IL18 (Fig. 1A). A central step in the formation of the NLRP3 inflammasome is the recruitment of ASC, the inflammasome adaptor protein, via the binding of the pyrin (PYD) domain (9). Immunofluorescence analysis of pancreas samples obtained from patients with PDAC (*n* = 4) showed increased expression and colocalization of NLRP3 with ASC compared with the pancreas of healthy individuals (*n* = 2; Fig. 1B). Furthermore, increased NLRP3 inflammasome formation, measured as ASC specks–like structure formation, was detected in PDAC samples compared with the normal tissue (Fig. 1C).

Next, we determined whether PDAC cells were expressing NLRP3 considering the increase observed in PDAC tissue. AsPC-1, MiaPaCa-2, and Panc-1 human PDAC cells were cultured for 24 hours and the spontaneous secretion of IL1β and IL18 were measured. As depicted in Supplementary Fig. S1A–S1D, *in vitro* these cells showed no production of these cytokines. Furthermore, intracellular analysis of NLRP3 expression showed that PDAC cells do not express this protein *in vitro* (Supplementary Fig. S1E). These data demonstrate increased NLRP3 expression and active inflammasome formation in PDAC biopsies.

### NLRP3 Activation Mediates IL1β and IL18 Production in PDAC *In Vitro*

**FIGURE 2:**
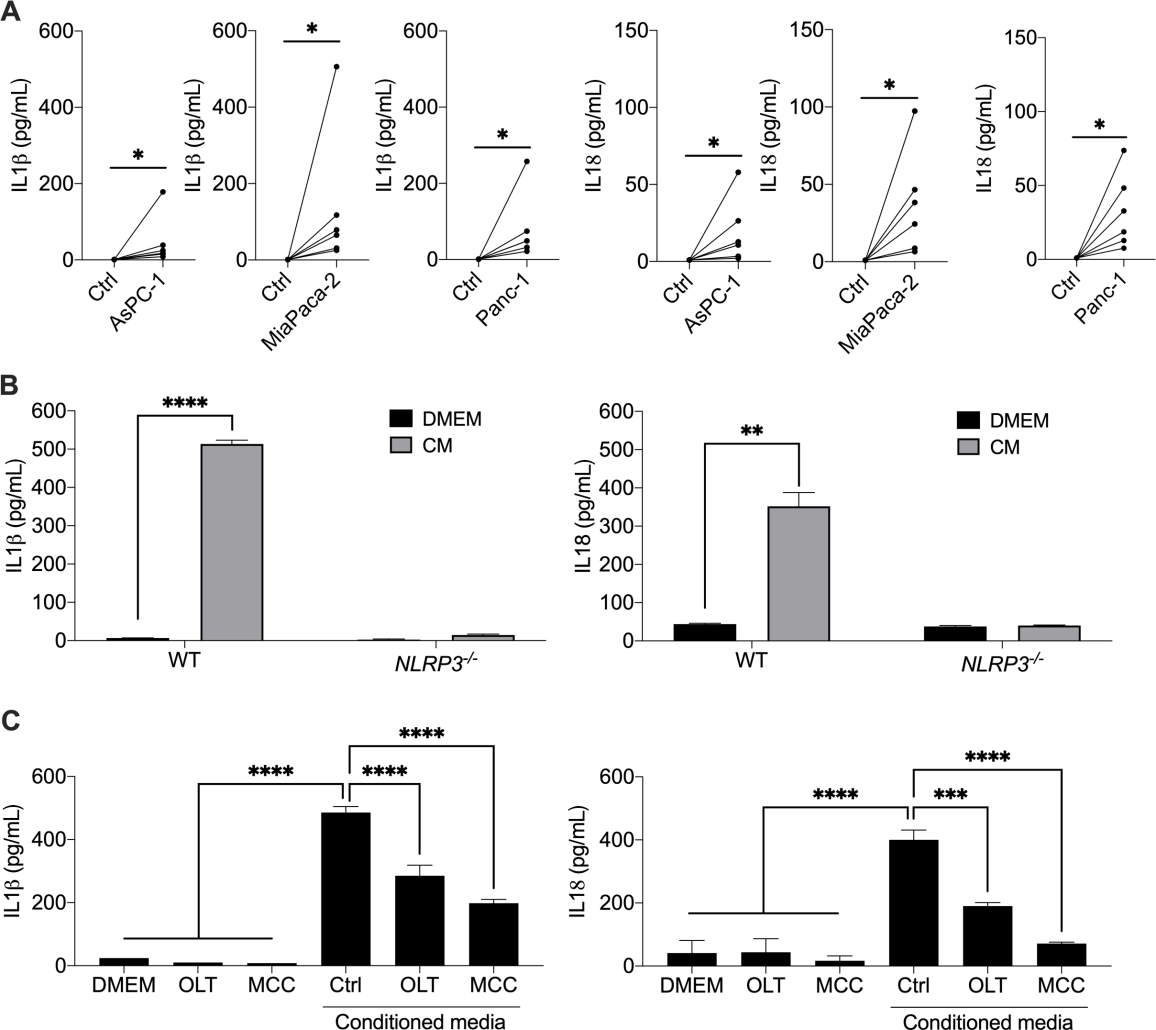
PDAC paracrine activity mediates NLRP3 activation. **A,** IL1β and IL18 secretion from freshly isolated PBMCs from healthy donors (*n* = 6) following stimulation with PDAC-conditioned media obtained from AsPC-1, MiaPaCa-2, and Panc-1 cells. **B,** IL1β and IL18 secretion from parenteral (WT) and NLRP3 deficient (NLRP3^−/−^) THP-1 cells following stimulation with PDAC-conditioned media obtained from Panc-1 cells (*n* = 3). **C,** IL1β and IL18 secretion from THP-1 cells following stimulation with PDAC-conditioned media obtained from Panc-1 cells in presence of the NLRP3 inhibitors OLT1177 (OLT) and MCC950 (MCC; *n* = 3). Cytokine levels were measured by specific ELISA kits. Data expressed as mean ± SEM; ****, *P* < 0.0001; **, *P* < 0.01; *, *P* < 0.05.

Next, we sought to determine whether PDAC cells release soluble factors that can induce NLRP3 activation in other cell types. Freshly isolated PBMCs from healthy donors (*n* = 6) were stimulated for 72 hours with soluble factors (CM) released by AsPC-1, MiaPaCa-2, and Panc-1. Stimulation of PBMCs with CM determined a significant increase in the levels of IL1β and IL18 in the supernatants (Fig. 2A). Next, we stimulated parenteral [wild-type (WT)] and NLRP3-deficient (*NLRP3*^−/−^) human leukemia monocytic (THP-1) cells with CM obtained from Panc-1 cells, as for Fig. 2A, to investigate a possible role of NLRP3 in the production of IL1β and IL18 in myeloid cells. As shown in Fig. 2B, CM significantly increased the release of IL1β and IL18 in WT THP-1 cells when compared with the unstimulated cells. In contrast, CM failed to induce the secretion of IL1β and IL18 in *NLRP3^−^^/^^−^* THP-1 cells (Fig. 2B). Similar results were obtained using two different NLRP3 inhibitors, OLT1177 (OLT) and MCC950 (MCC) in WT THP-1 cells (Fig. 2C). Altogether these data suggest that PDAC cells are able to stimulate IL1β and IL18 release in immune cells.

### NLRP3 Deletion or Pharmaceutical Blockage Reduces PDAC Progression

**FIGURE 3:**
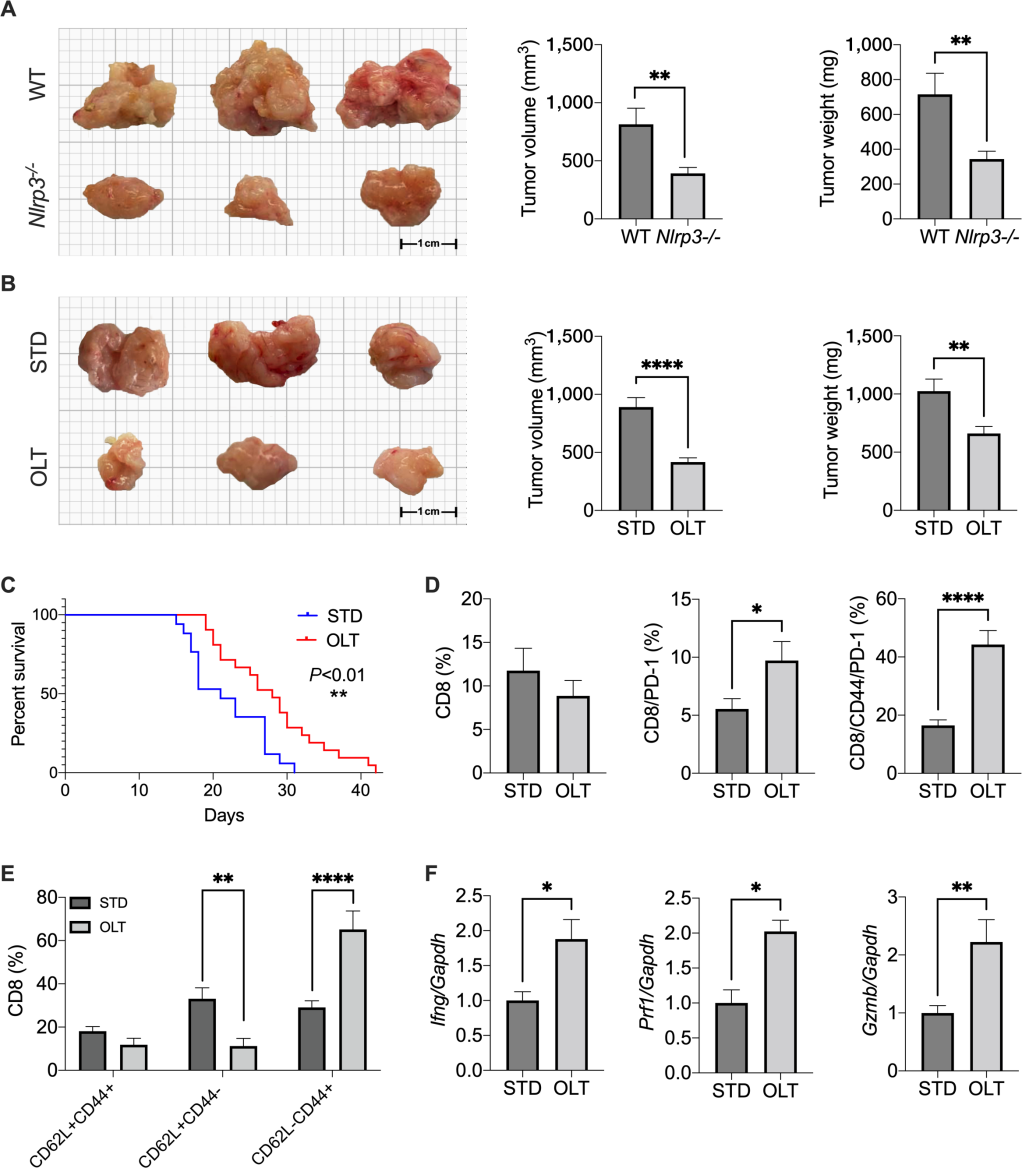
NLRP3 inhibition reduces PDAC progression. **A,** Tumor volume and weight in WT and *Nlrp3*^−/−^ mice (*n* = 8/group). **B,** Tumor volume and weight in mice fed standard (STD) or OL1177 (OLT) diet (*n* = 16/group). **C,** Survival curve in mice fed standard or OLT1177 diet (*n* = 20/group). **D,** Flow cytometry analysis of CD8, CD8/PD1, CD8/CD44/PD-1 cells in primary tumors of mice in B (*n* = 5/group). **E,** Flow cytometry analysis of memory (CD62L^+^/CD44^+^), naïve (CD62L^+^/CD44^−^), and effector (CD62L^−^/CD44^+^) CD8 cells in primary tumors of mice in D–F (*n* = 5/group). **F,** Gene expression of *Ifng*, *Prf1*, and *Gzmb* in primary tumors of mice in B (*n* = 5/group). Data expressed as mean ± SEM; ****, *P* < 0.0001; **, *P* < 0.01; *, *P* < 0.05.

To determine whether NLRP3 is required for PDAC progression *in vivo*, WT and NLRP3 (*Nlrp3^−^^/^^−^*) deficient mice were orthotopically implanted with FC1242 cells, a murine PDAC cell line derived from KPC mice (18). After 21 days from implantation, mice were sacrificed, and primary tumor dimensions and weight were measured. As depicted in Fig. 3A, lack of NLRP3 significantly reduced PDAC growth in mice. Next, we evaluated the effect of the NLRP3 inhibitor OLT1177 (19). Mice were implanted with FC1242 cells, as described above, and fed with OLT1177-enriched or standard diets. At sacrifice, tumor-bearing mice fed with OLT1177 showed a significant reduction in primary tumor mass and weight, similar to what was observed in the *Nlrp3^−^^/^^−^* mice (Fig. 3B). In addition, treatment with OLT1177 increased survival when compared with the mice treated with vehicle (Fig. 3C). To assure tumor NLRP3 was inhibited with OLT1177, the level of the inhibitor was measured in the mice. At sacrifice, the concentration of OLT1177 in primary tumors reached levels sufficient to prevent NLRP3 activation (Supplementary Fig. S2A and S2B; refs. 15, 20). NLRP3 inhibition following OLT1177 was further validated measuring active IL1β (p17) expression in the TME. As shown in Supplementary Fig. S2C and S2D, lower levels of p17 were detected in the primary tumors of mice treated with OLT1177. Cellular analysis of the primary tumors showed no significant changes in the infiltration of CD3^+^/CD8^+^ lymphocytes that could reflect smaller tumor growth (Fig. 3D). However, NLRP3 inhibition resulted in a significant increase in the infiltration of CD8^+^ cells expressing CD44 and PD-1 (Fig. 3D; Supplementary Fig. S3). Treatment with OLT1177 also showed an overall reduction in naïve (CD62L^+^/CD44^+^) and an increase in effector (CD62L*^−^*/CD44^+^) CD8 T cells when compared with control mice (Fig. 3E; Supplementary Fig. S3). Treatment with OLT1177 increased the levels of IFNγ (*Ifng*), perforin (*Prf1*), and granzyme B (*Gzmb*) in the primary tumors (Fig. 3F). When *Nlrp3*^−/−^ tumor-bearing mice were exposed to OLT1177, no differences in tumor growth and in the overall phenotype of CD4^+^ and CD8^+^ cells were observed against standard diet (Supplementary Fig. S4). Altogether, these data suggest that NLRP3 activation facilitates PDAC progression, and its inhibition increases CD8 T-cell activation.

### NLRP3 Inhibition Enhances Th1 Immunity

**FIGURE 4:**
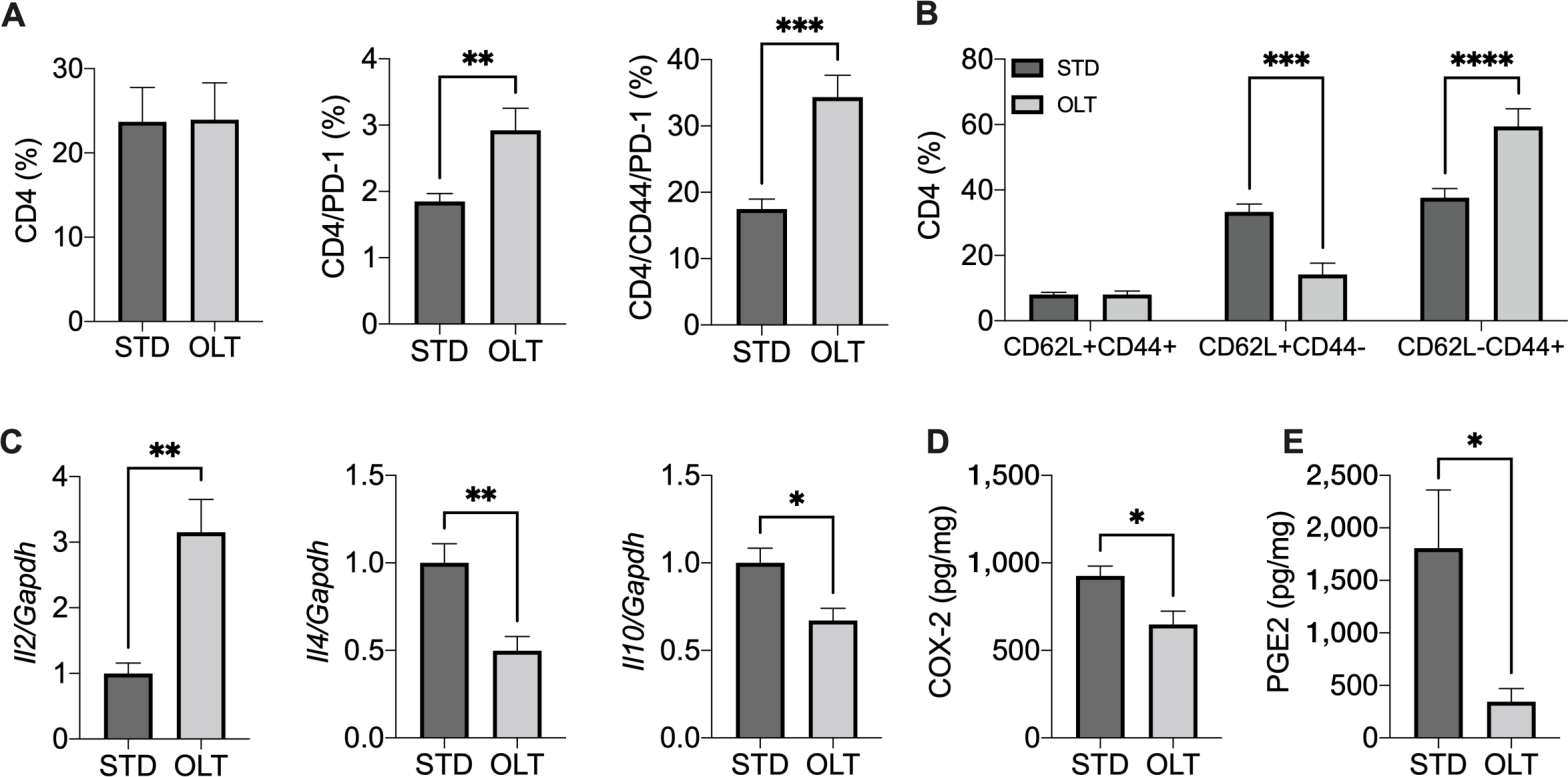
NLRP3 activation mediates a Th2-like inflammatory response. **A,** Flow cytometry analysis of CD4, CD4/PD1, CD4/CD44/PD-1 cells in primary tumors of mice fed standard (STD) and OLT1177 (OLT)-enriched diets (*n* = 5/group). **B,** Flow cytometry analysis of memory (CD62L^+^/CD44^+^), naïve (CD62L^+^/CD44^−^), and effector (CD62L^−^/CD44^+^) CD4 cells in primary tumors of mice fed STD and OLT-enriched diets (*n* = 5/group). **C,** Gene expression of *Il2*, *Il4*, and *Il10* in primary tumors of mice of mice fed STD and OLT-enriched diets (*n* = 5/group). **D,** COX-2 and PGE2 levels measured by ELISA in primary tumors of mice of mice fed STD and OLT-enriched diets (*n* = 5/group). **E,** PGE2 levels measured by ELISA in primary tumors of mice of mice fed STD and OLT-enriched diets (*n* = 5/group). Data expressed as mean ± SEM; ****, *P* < 0.0001; ***, *P* < 0.001; **, *P* < 0.01; *, *P* < 0.05.

Similar to what observed for CD3^+^/CD8^+^ lymphocytes, no significant changes in the infiltration of CD3^+^/CD4^+^ cells were observed in the primary tumors of mice treated with OLT1177 and vehicle (Fig. 4A). However, NLRP3 inhibition showed increased PD-1 expression in CD4 T cells and, more specifically, in the CD44 subpopulation (Fig. 4A). Furthermore, NLRP3 inhibition increased the effector/naïve CD4 ratio in the primary tumors (Fig. 4B). In the same samples, NLRP3 inhibition increased the levels of *Il2* while reducing the levels of *Il4* and *Il10* (Fig. 4C). These data suggest that NLRP3 activation mediates Th2 polarization of CD4 T cells, which has been shown to promote PDAC progression and reduce survival (21–23). Next, we measured the levels of cyclooxygenase-2 (COX-2), an inducible enzyme associated with immunosuppression and Th2 polarization in cancer (24–26) and known to be regulated by IL1β (27). Analysis of homogenized tumors showed a significant reduction in the levels of COX-2 following treatment with OLT1177 (Fig. 4D). This reduction mirrored a decrease in the levels of prostaglandin E2 (PGE2), a potent immunosuppressor for T-cell activity (Fig. 4E).

Considering the increased expression of PD-1 on T cells following NLRP3 inhibition, we determined whether such expression was the result of an earlier activation of these cells or an indication that T cells were progressively losing effector function in response to persistent activation (28, 29). Therefore, we investigated the T cell phenotype in the presence or absence of OLT1177 at 2 weeks from FC1242 cells implantation, a week earlier compared with the previous study design. As shown in Supplementary Fig. S5A and S5B, NLRP3 inhibition with OLT1177 showed a reduction in tumor volume and weight at 2 weeks from KPC implantation. Similar to what was observed at 21 days, NLRP3 inhibition did not show changes in T-cell influx in the primary tumor (Supplementary Fig. S5C); however, increased PD-1 expression was observed (Supplementary Fig. S5C). These data, in combination with the increase of T-cell activation markers measured at 3 weeks, suggest that NLRP3 inhibition sustains T-cell activation when compared with the control group.

**FIGURE 5:**
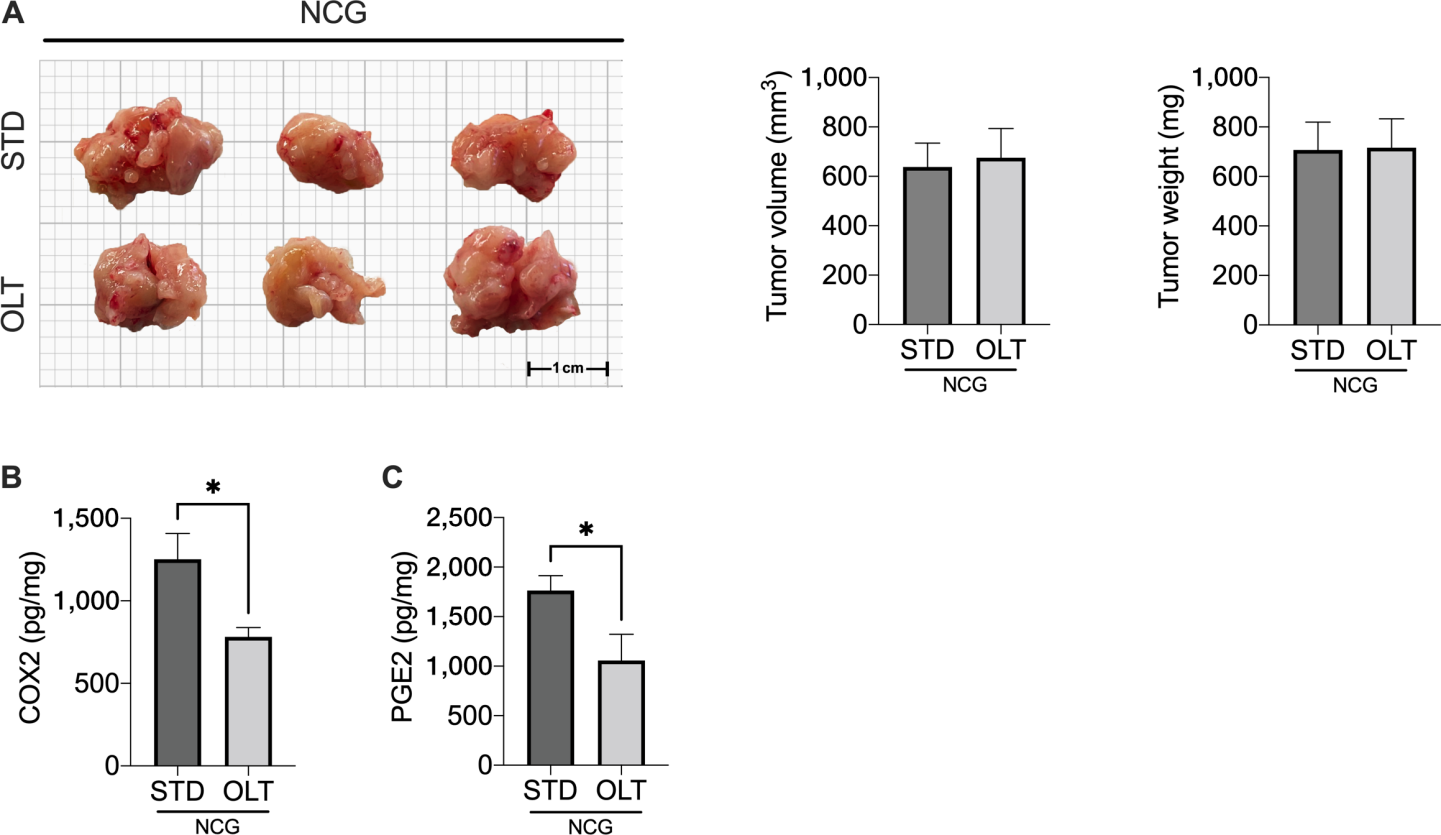
NLRP3 activation mediates lympthocyte suppression. **A,** Tumor volume and weight in NCG mice fed standard (STD) or OL1177 (OLT) diet (*n* = 5/group). **B,** COX-2 levels measured by ELISAs in primary tumors of mice of mice fed STD and OLT-enriched diets. **C,** PGE2 levels measured by ELISAs in primary tumors of mice of mice fed STD and OLT-enriched diets. Data expressed as mean ± SEM. *, *P* < 0.05.

Next, we determined whether the reduction in tumor growth following NLRP3 inhibition was T-cell dependent. NCG mice were implanted with FC1242 cells and treated with OLT1177-enriched or standard diets. As shown in Fig. 5A, there was no difference in tumor growth between NCG mice fed standard or OLT1177 diets. Nevertheless, OLT1177-treated mice showed a significant reduction in COX-2 and PGE2, consistent with that observed in the WT mice (Fig. 5B and C). Altogether, these data demonstrate that NLRP3 inhibition restored an effective Th1-like response which ultimately increased cytotoxic CD8 T-cells activation.

### CSF-1 Receptor Engagement Activates NLRP3

**FIGURE 6:**
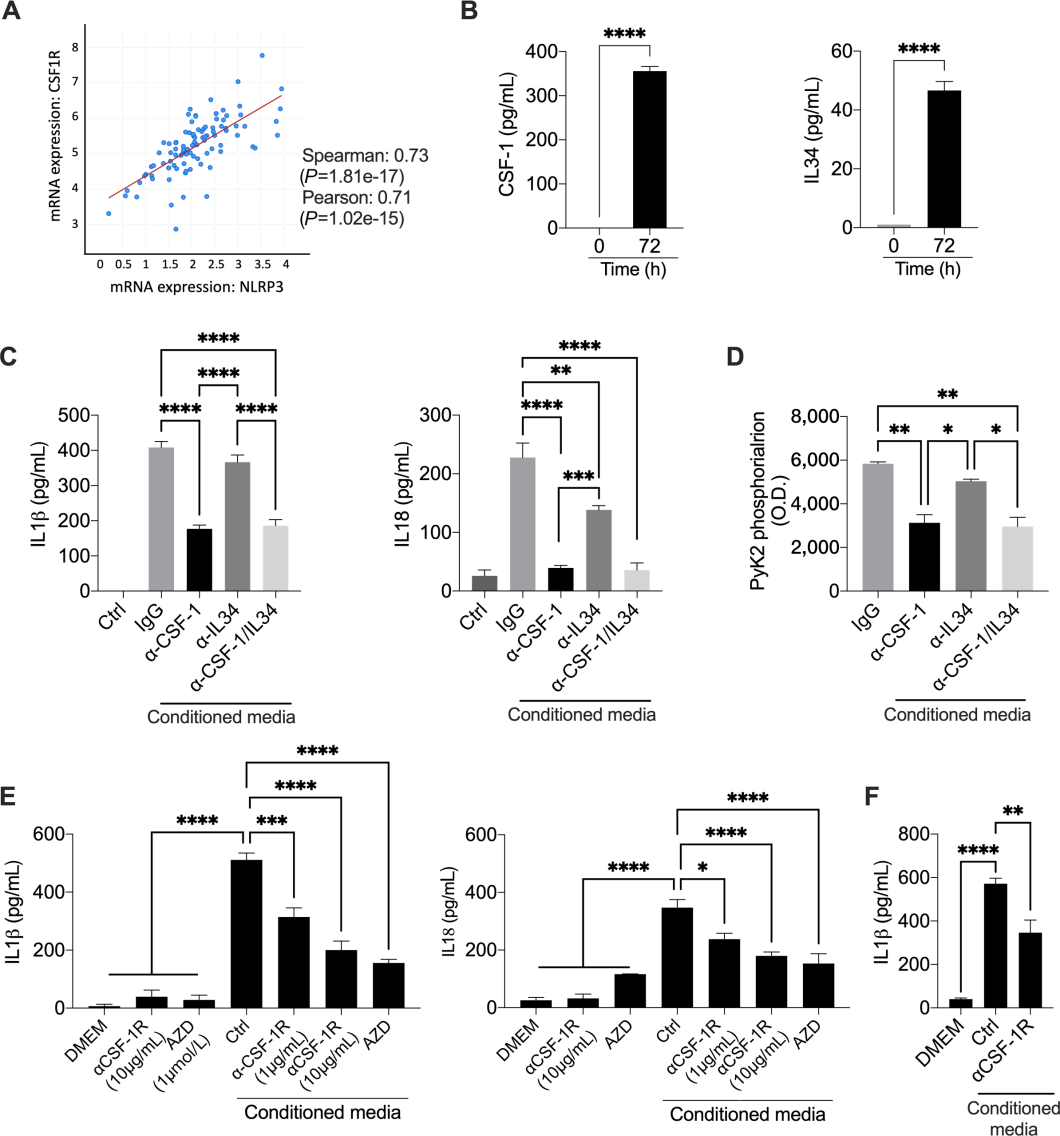
CSF-1R engagement induces IL1β and IL18 release. **A,** Correlation between CSF-1R and NLRP3 expression in PDAC (*n* = 96) from TGCA dataset. **B,** Spontaneous CSF-1 and IL34 secretion in Panc-1 cells at 72 hours (*n* = 3). **C,** IL1β and IL18 secretion in THP-1 cells following stimulation with PDAC-conditioned media obtained from Panc-1 cells in presence of IgG (control), α-CSF-1, α-IL34, α-CSF-1/IL34 (*n* = 3). **D,** Phosphorylation levels of PyK2 (Tyrosine 402) in THP-1 stimulated in D and E (*n* = 3). **E,** IL1β and IL18 secretion from THP-1 cells following stimulation with PDAC-conditioned media obtained from Panc-1 cells in presence of α-CSF-1R and an CSF-1R inhibitor (AZD; *n* = 3). **F,** IL1β secretion from freshly isolated PBMCs from healthy donors (*n* = 4) following stimulation with PDAC-conditioned media obtained from Panc-1 cells in presence of α-CSF-1R. Cytokine levels were measured by specific ELISAs. Data expressed as mean ± SEM; ****, *P* < 0.0001; ***, *P* < 0.001; **, *P* < 0.01; *, *P* < 0.05.

To better understand the mechanism behind NLRP3 activation in PDAC, we investigated how PDAC cells were able to stimulate its induction. TGCA analysis of 96 patients with PDAC (30) showed a significant correlation (Spearman: 0.73; *P* = 1.81e^−17^) for *NLRP3* and *CSF1R* (Fig. 6A). Considering that CSF-1 receptor (CSF-1R) has two ligands, CSF-1 and IL34 (31), we measured their spontaneous production using Panc-1 cells to determine which ligand was more relevant for NLRP3 activation. As shown in Fig. 6B, Panc-1 cells spontaneously secrete both cytokines; however, at 72 hours of culture, the overall levels of CSF-1 were much higher compared with the one of IL34. To further explore the contribution of CSF-1 and IL34 in IL1β and IL18 production, we stimulated THP-1cells with conditioned media (as previously described in Fig. 2) where CSF-1, IL34, or both were neutralized using specific antibodies. As shown in Fig. 6C stimulation of THP-1 with conditioned media with α-CSF-1, α-IL34, and α-CSF-1/IL34 showed a reduction in IL1β and IL18 production by all three conditions when compared with the cell stimulated with the control CM (IgG). Notably, CM with α-CSF-1 showed greater inhibition when compared with α-IL34, similarly of what measured in the THP-1 cells treated CM with α-CSF-1/IL34 (Fig. 6C). Mechanistically, we observed a 47% reduction in the phosphorylation of the tyrosine kinase PyK2 on the Tyrosine 402 following THP-1 stimulation with CM+αCSF-1, while stimulation of THP-1 cells with CM+αIL34 resulted in a more modest reduction (14%; Fig. 6D). PyK2 regulates multiple signaling events crucial for myeloid cells morphology, activation, and migration (32) and, it has been reported to be activated following CSF-1R stimulation (33, 34). Relevant to NLRP3, it has been shown that PyK2 is required for the correct assembly of the NLRP3 inflammasome (35, 36). In conclusion, albeit other pathways cannot be excluded, engagement of CSF-1R with both ligands induced NLRP3 activation. However, our data suggest that the contribution of CSF-1 to this pathway is more relevant when compared with IL34.

Then, we validated these data blocking directly the CSF-1R using a neutralizing antibody and a small selective molecule (AZD7507). As described previously, THP-1 cells were stimulated for 72 hours with CM from Panc-1 cells. As shown in Fig. 6E, inhibition of the CSF-1R resulted in a significant reduction in the production of IL1β and IL18 when compared to the cells in control. A similar reduction in IL1β was also observed in freshly isolated PBMCs (Fig. 6F).

**FIGURE 7:**
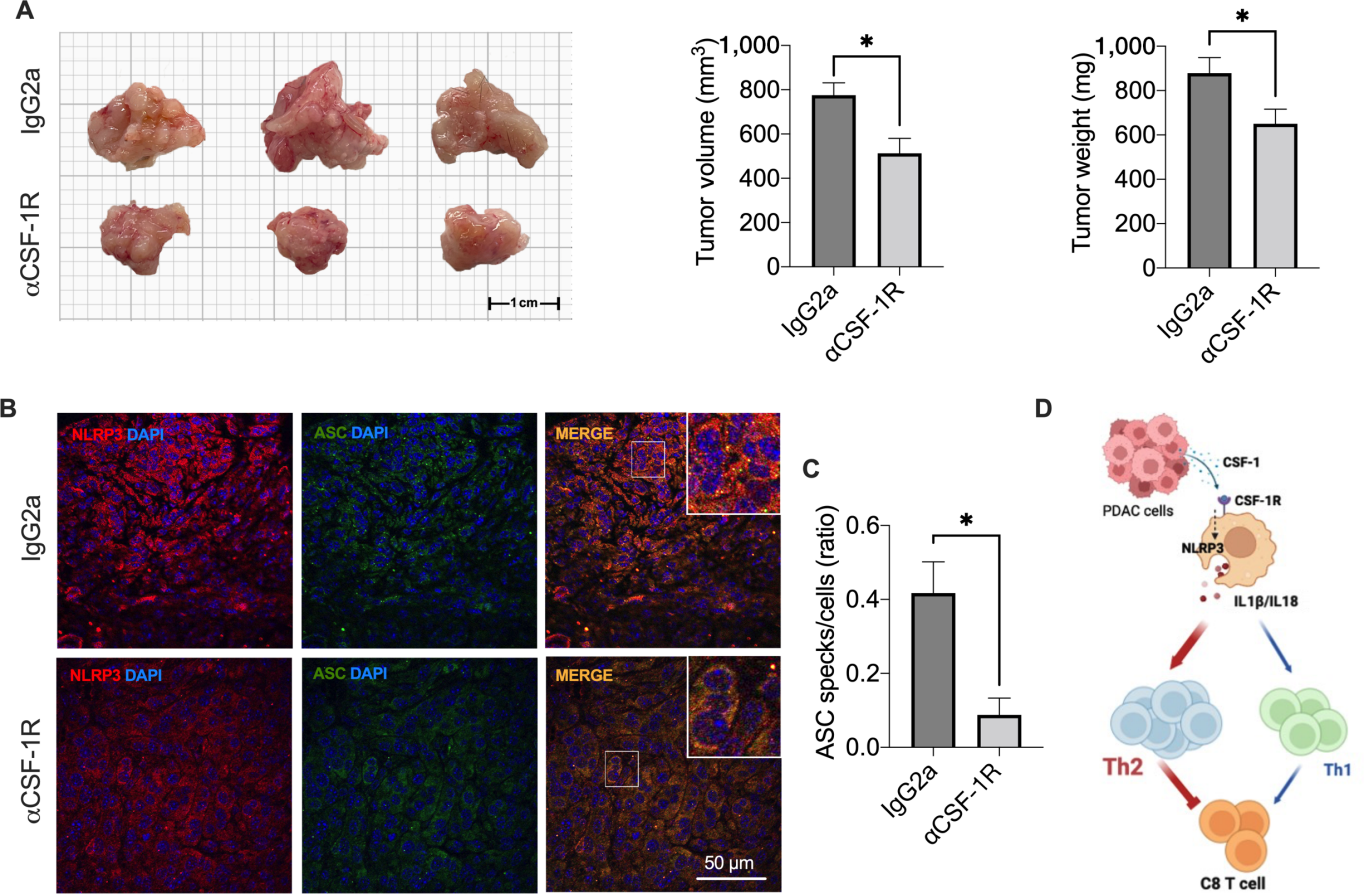
CSF-1R engagement induces NLRP3 activation *in vivo*. **A,** Tumor volume and weight in mice treated with α-CSF-1R or matching IgG (*n* = 6/group). **B,** Immunofluorescence staining from NLRP3 (red) and ASC (green) in primary tumors treated with α-CSF-1R or matching IgG (*n* = 3/group). **C,** Quantification of speck-like structure in mice treated with α-CSF-1R or matching IgG (*n* = 3/group). **D,** Schematic graphic representation of the proposed NLRP3 activation and signaling in PDAC (created with BioRender.com).

Next, we determined whether CSF-1 signaling was relevant for NLRP3 activation and NLRP3 inflammasome formation *in vivo*. Using the same orthotopic FC1242 model described before, mice were treated with anti-CSF-1R or a matching concentration of IgG2a. As shown in Fig. 7A, mice treated with the neutralizing antibody showed a significant reduction in tumor growth and weight. Analysis of the primary tumors revealed a phenotype of CD4 and CD8 T cells in the mice treated with the α-CSF-1R comparable with the one observed in mice treated with the NLRP3 inhibitor OLT1177 (Supplementary Fig. S6A and S6B), suggesting a similar mechanism between the two treatments. Notably, tumor-bearing mice treated with anti-CSF-1R showed a reduction in the colocalization of NLRP3 and ASC (Fig. 7B) that resulted in a significant decrease in the formation of ASC specks (Fig. 7C). Consistently, mice treated with anti-CSF-1R also showed a reduction in the processed form of IL1β (p17) in the tumors (Supplementary Fig. S6C and S6D). In conclusion, these data demonstrate that PDAC cells mediate NLRP3 activation via CSF-1R engagement (Fig. 7D).

### NLRP3 Inhibition Increases the Efficacy of Gemcitabine

**FIGURE 8:**
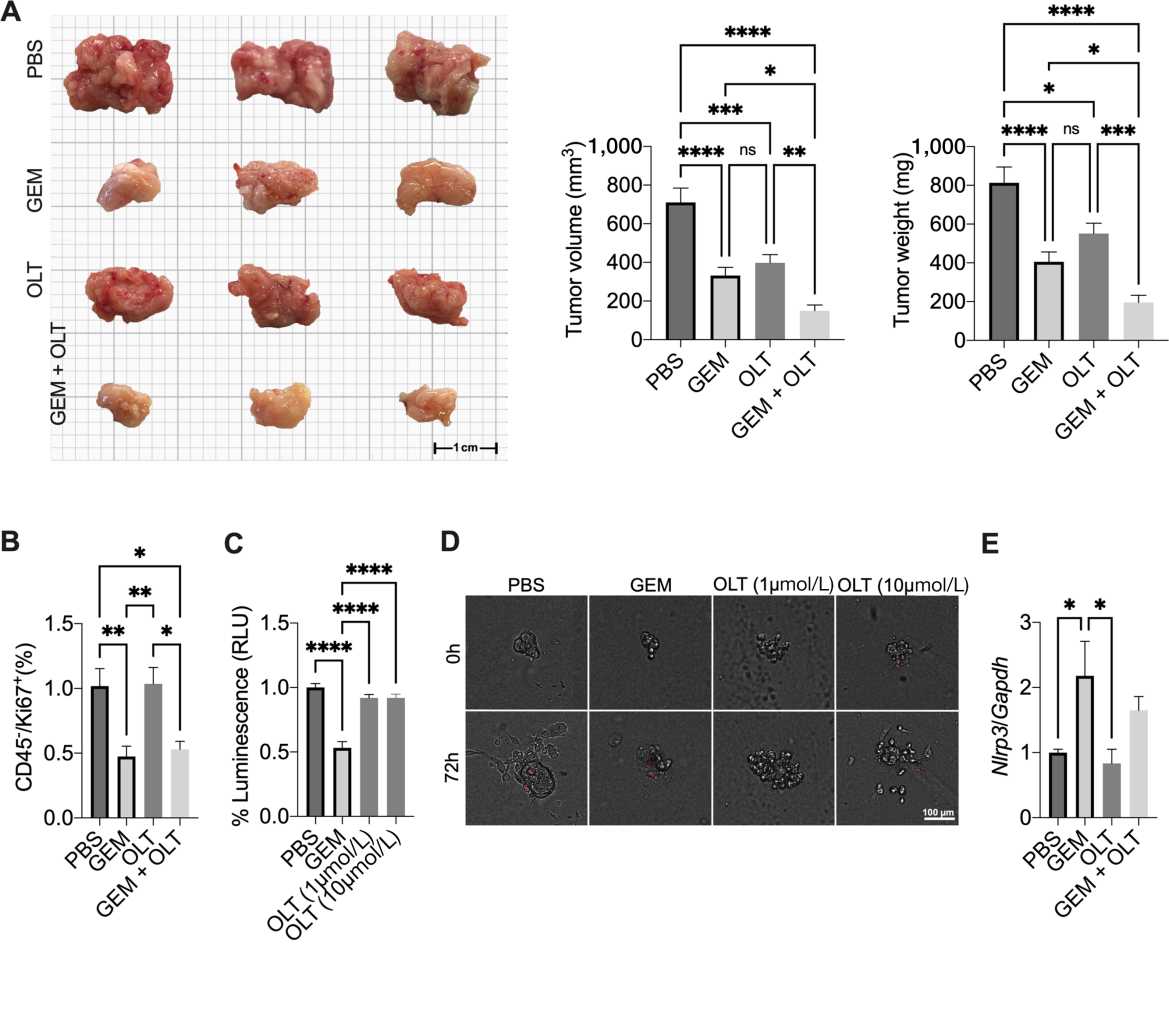
NLRP3 inhibition increases gemcitabine efficacy. **A,** Tumor volume and weight in mice treated with vehicle (PBS), gemcitabine (GEM), OLT1177 (OLT), and GEM+OLT (*n* = 8/group). **B,** Flow cytometry analysis of Ki67 expression in CD45^−^ cells from the primary tumors obtained in A (*n* = 8/group). **C,** Viability of human PDAC organoids after 72 hours of culture in presence of gemcitabine (GEM) or OLT1177 (OLT; *n* = 3). **D,** Representative images of organoids from C. **E,** NLRP3 expression in primary tumors obtained in A (*n* = 3/group). Data expressed as mean ± SEM; ****, *P* < 0.0001; ***, *P* < 0.001; **, *P* < 0.01; *, *P* < 0.05.

Next, we evaluated whether NLRP3 inhibition in combination with gemcitabine would increase the efficacy of the single-agent chemotherapy. Tumor-bearing mice were treated with vehicle, gemcitabine, OLT1177, and gemcitabine+OLT1177. Expectedly gemcitabine alone induced a significant reduction in tumor volume and weight when compared with the mice in control (Fig. 8A). Mice treated with OLT1177 also showed a reduction in tumor growth versus vehicle (Fig. 8A). No significant differences were observed in tumor growth between gemcitabine and OLT1177 alone (Fig. 8A). Mice treated with gemcitabine and OLT1177 showed a significant reduction in tumor growth compared with the vehicle-treated mice and versus the respective monotherapy (gemcitabine and OLT1177) as shown in Fig. 8A. In the primary tumors, analysis of Ki67 in CD45-negative cells showed a reduction of this marker following gemcitabine treatment (Fig. 8B). No significant changes in cells proliferation were observed following OLT1177 when compared with vehicle-treated mice (Fig. 8B). Mice treated with gemcitabine+OLT1177 combination showed a significant reduction in Ki67 when compared with vehicle- and OLT1177-treated mice (Fig. 8B). When human PDAC organoids were treated with gemcitabine, a significant reduction in cell viability was measured (Fig. 8C and D). No changes in viability were observed when PDAC organoids were treated in presence of either 1 or 10 µmol/L OLT1177 compared with the cells cultured in absence of the inhibitor (Fig. 8C and D). Analysis of NLRP3 expression following gemcitabine treatment showed that the chemotherapy agent increased NLRP3 expression (Fig. 8E). Altogether these data demonstrate that the addition of NLRP3 inhibition with OLT1177 to the standard-of-care gemcitabine potentiates the efficacy of gemcitabine alone. It further suggests that gemcitabine increases NLRP3 expression in the TME.

## Discussion

PDAC is one of the most lethal cancers that invariably presents at an advanced stage and is refractory to most treatment modalities (37, 38). Current drug regimens are not curative and highly debilitating keeping patients at high risk for progression. Notably, although the biological basis of PDAC clearly involves immune tolerance (39), no therapy developed to date is actually designed to increase the efficiency of the immune system to control PDAC cell expansion. It has been shown that the recruitment and activation of inflammatory cells such as macrophages play a key role in the pathogenesis of pancreatic cancers (3, 40). Thus, identification of innate immune signaling(s) that participate in the myeloid-mediated immunosuppression could represent an effective strategy to reverse T-cell suppression.

In this study, we characterized a key role for NLRP3 in PDAC progression and we elucidate the mechanism by which PDAC cells mediate NLRP3 activation. We show that NLRP3 is highly expressed in pancreatic cancer tissue when compared with the normal pancreas. NLRP3 is a cytosolic receptor that, following activation, determines the maturation of the biological inactive inflammatory cytokines IL1β and IL18 into their biologically active forms (9). We were able to demonstrate the formation of the NLRP3 inflammasome in human PDAC samples. Consistently, IL1β levels were found elevated in PDAC samples (6). Notably, recent studies have reported a positive correlation between NLRP3 expression and reduced survival in patients with PDAC, reinforcing the hypothesis of an important role for NLRP3 in PDAC progression (13). In humans, 14 members of the NLRP family have been identified (41), herein we demonstrated that NLRP3 mediates the increase in IL1β production in PDAC; however, the activation of other members of the family was not investigated in this study; therefore, it cannot be excluded.

In an orthotopic PDAC murine model, genetic and pharmacologic inhibition of NLRP3 significantly reduced tumor growth and weight when compared with the tumor-bearing mice in control. Analysis of the primary tumors revealed no differences in the overall infiltration of T cells (CD4 and CD8) however, a change in their phenotype was detected. A significant increase of effector T cells was observed with the small and orally active NLRP3 inhibitor OLT1177 (rINN dapansutrile; ref. 19). T cells were also characterized by a significant increase in PD-1 expression when compared with the cells in control. Increased PD-1 expression in T cells reflects activation but it is also associated with cell exhaustion (28, 29, 42). To determine whether NLRP3 inhibition caused an early activation of these cells, we looked at CD4 and CD8 cells at 14 days, 7 days prior to the usual sacrifice. At 14 days, we observed a comparable phenotype, suggesting a prolonged activation of T cells in the presence of NLRP3 inhibition. This conclusion was supported by the increased expression of CD8 T cell activity markers such as IFNγ, perforin, and Gzmb. Furthermore, we found that NLRP3 inhibition determined a Th1 polarization as measured by the intratumoral increase in IL2 while reducing Th2 response in the TME as shown by the reduction in IL10 and IL4. Overall, these data support an augmented activation of CD8 T cells in the presence of the NLRP3 inhibitor OLT1177. This is particularly relevant in the context of PDAC progression as it has been shown that increased Th1/Th2 ratio is an independent predictive factor of survival after surgery in patients with PDAC and that Th2-type inflammation is associated with reduced survival (21–23).

A critical finding of this study was that PDAC cells exploit NLRP3 activation via the engagement of CSF-1R. CSF-1R expression has been associated with poor prognosis and its inhibition has shown very promising preclinical results (43, 44). Albeit these findings established the rationale for clinical testing, the outcomes of these clinical trials did not show significant therapeutical benefits (45). The reported accumulation of polymorphonuclear myeloid-derived suppressor cells (PMN-MDSCs) following CSF-1R inhibition could explain the therapeutic limitations observed with CSF-1R inhibitors (46). Notably, inhibition of NLRP3 has shown not to increase these cells in PDAC models and to reduce both monocytic MDSCs and PMN-MDSCs in other cancers such as melanoma and lung carcinoma (12, 15, 47).

Considering the limited efficacy of the current treatments for PDAC is becoming a pressing issue to find combinatory strategies to treat human pancreatic cancer. In our study, we show that the combination of the human-safe NLRP3 inhibitor OLT1177 (48, 49) with gemcitabine significantly reduced tumor progression when compared with the monotherapies. Gemcitabine is a chemotherapy that prevents DNA synthesis halting the expansion of highly proliferating cells. Our studies show that NLRP3 inhibition reduces the PDAC-associated immunosuppression without affecting tumor cells proliferation. Mechanistically, treatment with OLT1177 showed no effect on non-immune cells proliferation but determined a significant reduction in COX2/PGE2 signaling. The independent targets of gemcitabine and OLT1177 provide the basis for the additive effect of the combinational therapy and could support the rationale for further clinical implications. Interestingly, analysis of 60 patients with advanced pancreatic cancer who received gemcitabine showed that the serum levels of IL1β and IL6 were poor prognostic factors for overall survival and the levels of these cytokines predicted the efficacy of gemcitabine in this population (7).

In conclusion, this study shows that inhibition of NLRP3 prevents the generation of the PDAC permissive inflammation reconstituting an effective cytotoxic adaptive immunity. It also supports the rationale for combinational strategies that could provide long-term and tolerable treatments for patients with pancreatic cancer.

## Supplementary Material

Supplementary Figure S1 Level of NLRP3, IL 1b and IL 18 in PDAC cells.

Supplementary Figure S2 OLT1177 levels in mice.

Supplementary Figure S3 Gating strategy used for the definition of the populations of interest.

Supplementary Figure S4 Comparison of tumor growth in Nlrp3-/- mice treated with OLT1177

Supplementary Figure S5 NLRP3 inhibition reduces PDAC progression

Supplementary Figure S6 Effect of anti-CSF-1R on T cells
