# Supplementary material for: Pancreatic Ductal Adenocarcinoma Cells Regulate NLRP3 Activation to Generate a Tolerogenic Microenvironment
Source: Cancer Res Commun. 2023 Sep 20;3(9):1899–911. doi: 10.1158/2767-9764.CRC-23-0065 (PMC10510589; doi:10.1158/2767-9764.CRC-23-0065)
Supplement: Supplementary Figure S1 — Level of NLRP3, IL 1b and IL 18 in PDAC cells. [file crc-23-0065-s01.docx]

**Supplementary Figure S1**


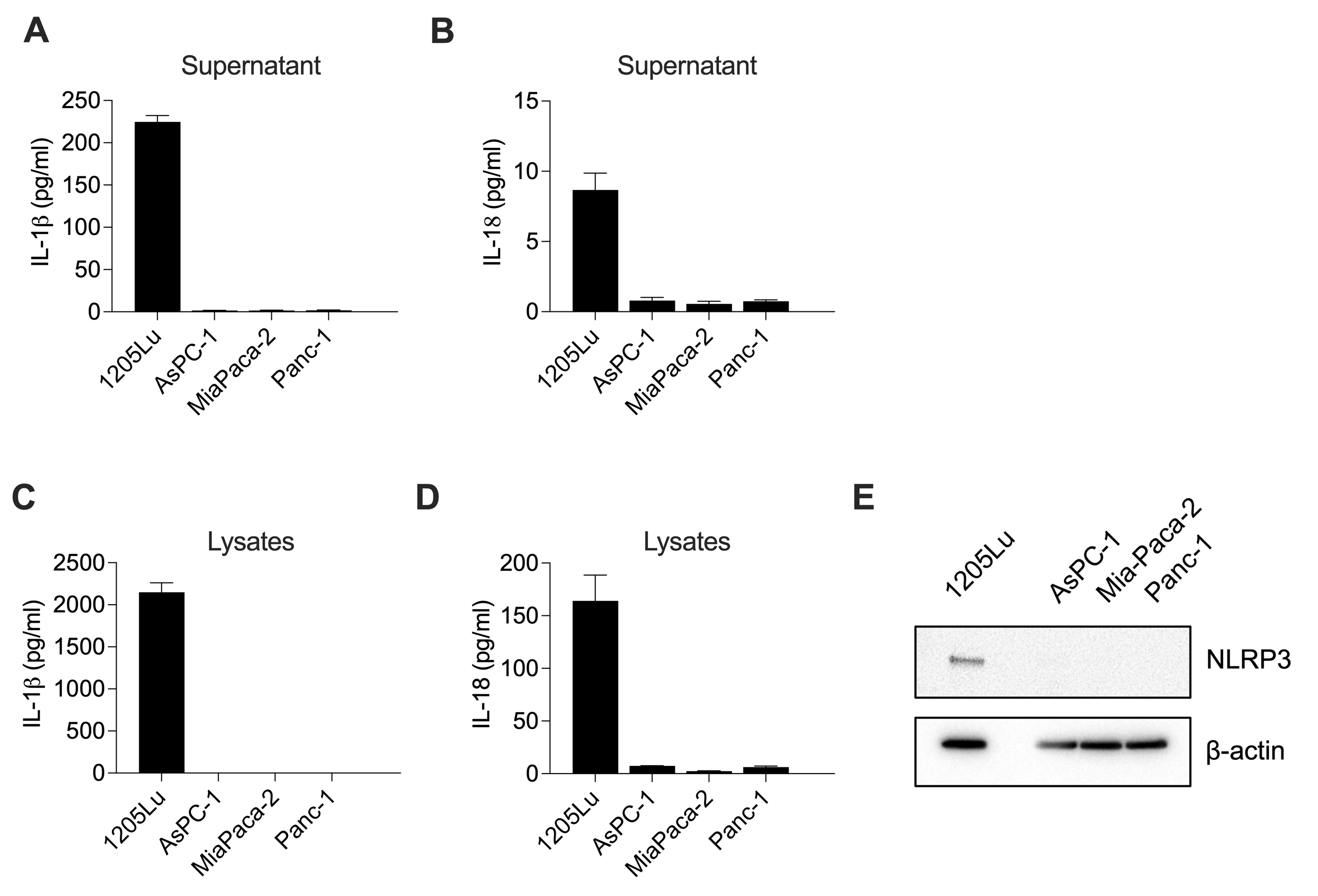


**Level of NLRP3, IL‑1β and IL‑18 in PDAC cells.** (**A**) Spontaneous release of IL‑1β in 1205Lu (melanoma), AsPC-1, MiaPaCa-2, and Panc-1 cells at 24 hours of culture (n=3). (**B**) Spontaneous release of IL‑18 in 1205Lu (melanoma), AsPC-1, MiaPaCa-2, and Panc-1 cells at 24 hours of culture (n=3). (**C**) Intracellular level of IL‑1β in 1205Lu (melanoma), AsPC-1, MiaPaCa-2, and Panc-1 cells at 24 hours of culture (n=3). (**D**) Intracellular level of IL‑18 in 1205Lu (melanoma), AsPC-1, MiaPaCa-2, and Panc-1 cells at 24 hours of culture (n=3). (**E**) NLRP3 expression in 1205Lu (melanoma), AsPC-1, MiaPaCa-2, and Panc-1 cells at 24 hours of culture. 1205Lu melanoma cells were used as positive control.
