# Supplementary material for: Pancreatic Ductal Adenocarcinoma Cells Regulate NLRP3 Activation to Generate a Tolerogenic Microenvironment
Source: Cancer Res Commun. 2023 Sep 20;3(9):1899–911. doi: 10.1158/2767-9764.CRC-23-0065 (PMC10510589; doi:10.1158/2767-9764.CRC-23-0065)
Supplement: Supplementary Figure S2 — OLT1177 levels in mice. [file crc-23-0065-s02.docx]

**Supplementary Figure S
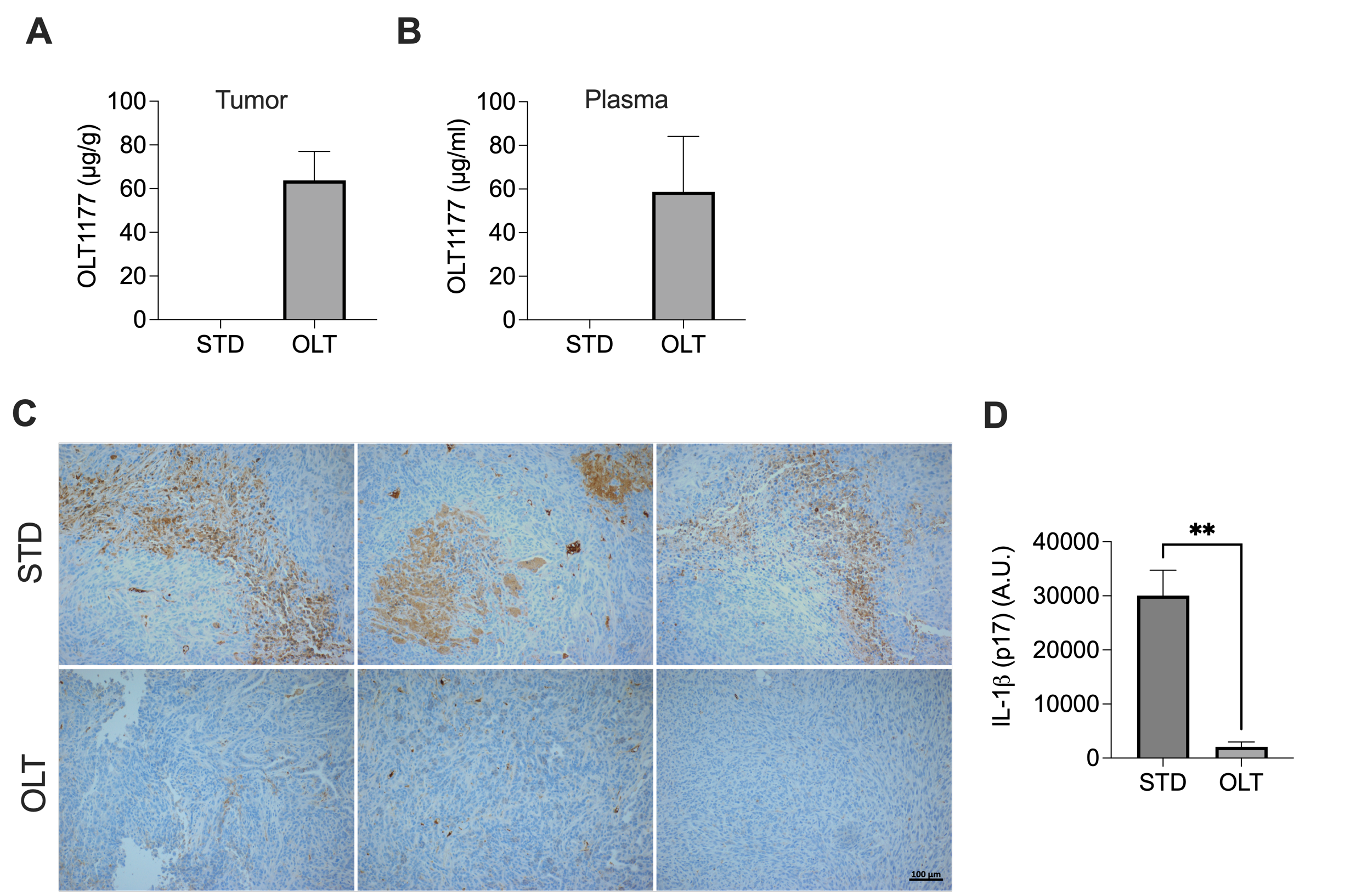
2**

**OLT1177 levels in mice.** Tumor (**A**) and plasma (**B**) OLT1177 levels measured in PDAC-bearing mice following three weeks of treatment (n=6/group). (**C**) Immunohistochemistry for active IL-1β (p17) levels in PDAC tumors of mice treated with vehicle (STD, top panel) or OLT1177 (OLT, bottom panel). Each panel represents a single mouse. (**D**) Immunoreactivity of processed IL‑1β expression (n=3/group). Data expressed as mean ± SEM, **P<0.01.
