# Supplementary material for: Pancreatic Ductal Adenocarcinoma Cells Regulate NLRP3 Activation to Generate a Tolerogenic Microenvironment
Source: Cancer Res Commun. 2023 Sep 20;3(9):1899–911. doi: 10.1158/2767-9764.CRC-23-0065 (PMC10510589; doi:10.1158/2767-9764.CRC-23-0065)
Supplement: Supplementary Figure S3 — Gating strategy used for the definition of the populations of interest. [file crc-23-0065-s03.docx]

**Supplementary Figure S3**


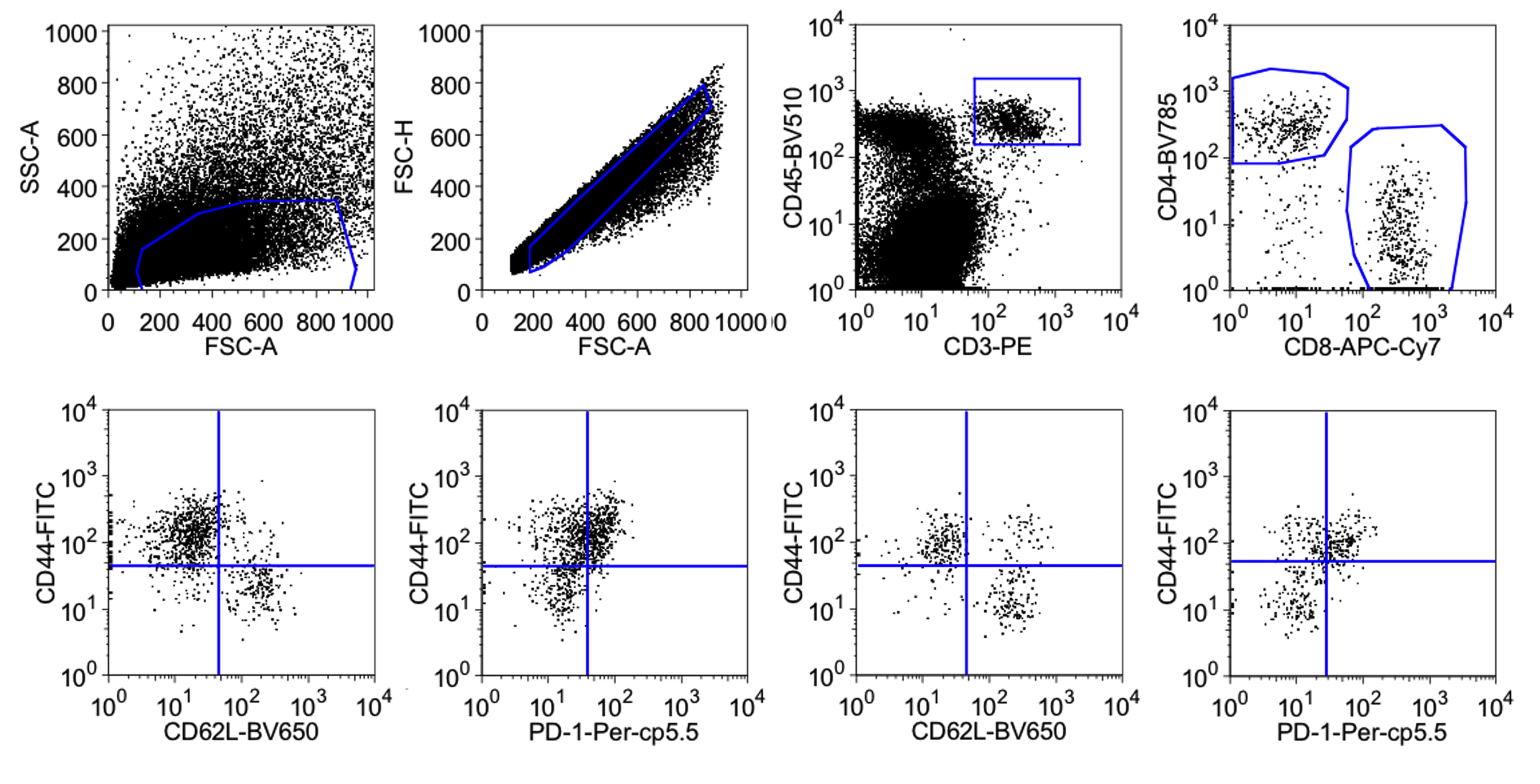


**Gating strategy used for the definition of the populations of interest.** Representative schematic of the used gating strategy to identify cells based on the reported membrane markers. Prior to CD45/CD3 gating, live cell gating was performed using propidium iodide.
