# Supplementary material for: Pancreatic Ductal Adenocarcinoma Cells Regulate NLRP3 Activation to Generate a Tolerogenic Microenvironment
Source: Cancer Res Commun. 2023 Sep 20;3(9):1899–911. doi: 10.1158/2767-9764.CRC-23-0065 (PMC10510589; doi:10.1158/2767-9764.CRC-23-0065)
Supplement: Supplementary Figure S4 — Comparison of tumor growth in Nlrp3-/- mice treated with OLT1177 [file crc-23-0065-s04.docx]

**Supplementary Figure S4**

**
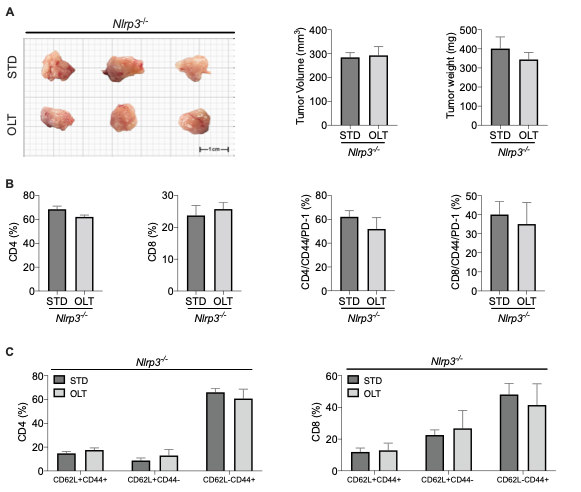
**

**Comparison of tumor growth in Nlrp3^-/-^ mice treated with OLT1177.** (**A**) Tumor volume and weight in Nlrp3^-/-^ mice fed standard (STD) or OL1177 (OLT) diet (n=5/group). (**B**) Flow cytometry analysis of CD4, CD8, CD4/CD44/PD1, CD8/CD44/PD-1 cells in primary tumors of mice in A. (**C**) Flow cytometry analysis of memory (CD62L+/CD44+), naïve (CD62L+/CD44-) and effector (CD62L-/CD44+) CD4 and CD8 cells in primary tumors of mice in A-C (n=5/group).
