# Supplementary material for: Pancreatic Ductal Adenocarcinoma Cells Regulate NLRP3 Activation to Generate a Tolerogenic Microenvironment
Source: Cancer Res Commun. 2023 Sep 20;3(9):1899–911. doi: 10.1158/2767-9764.CRC-23-0065 (PMC10510589; doi:10.1158/2767-9764.CRC-23-0065)
Supplement: Supplementary Figure S5 — NLRP3 inhibition reduces PDAC progression [file crc-23-0065-s05.docx]

**Supplementary Figure S5**

**NLRP3 inhibition reduces PDAC progression.** (**A**) Tumor volume in mice fed standard (STD) or OL1177 (OLT) diet at 14 days post-tumor implantation (n=10/group). (**B**) Tumor weight in mice fed standard (STD) or OL1177 (OLT) diet at 14 days post-tumor implantation (n=10/group). (**C**) Flow cytometry analysis of CD4, CD8 and CD8/CD44/PD-1 cells in primary tumors of mice in A and B. Data expressed as mean ± SEM, **P < 0.01, *P <0.05.
