# Supplementary material for: Pancreatic Ductal Adenocarcinoma Cells Regulate NLRP3 Activation to Generate a Tolerogenic Microenvironment
Source: Cancer Res Commun. 2023 Sep 20;3(9):1899–911. doi: 10.1158/2767-9764.CRC-23-0065 (PMC10510589; doi:10.1158/2767-9764.CRC-23-0065)
Supplement: Supplementary Figure S6 — Effect of anti-CSF-1R on T cells [file crc-23-0065-s06.docx]

**Supplementary Figure S6**

**Effect of α-CSF-1R on T cells.** (**A**) Flow cytometry analysis of CD4, CD8, CD4/CD44/PD-1, and CD8/CD44/PD-1 cells in primary tumors of mice treated with α-CSF-1R and a matching volume of IgG (n=6/group). (**B**) Flow cytometry analysis of memory (CD62L+/CD44+), naïve (CD62L+/CD44-) and effector (CD62L-/CD44+) CD4 and CD8 cells in primary tumors of mice treated with α-CSF-1R and a matching volume of IgG (n=6/group). (**C**) Immunohistochemistry for processed IL-1β (p17) levels in PDAC tumors following treated with IgG (top panel) or α-CSF-1R (bottom panel). Each panel represents a single mouse. (**D**) Immunoreactivity of processed IL‑1β expression (n=3/group). Data expressed as mean ± SEM, ***P < 0.001, **P < 0.01, *P < 0.05.
